# Physicochemical Comparison of Hydroxyapatite Extracted from Adult and Young Duroc-Jersey Porcine Bones: Influence of Predominant Ionic Composition on Enhanced Potential Applications

**DOI:** 10.1021/acsomega.6c06190

**Published:** 2026-08-12

**Authors:** Leon R. Bernal-Alvarez, Beatriz Marcela Millán Malo, Mario E. Rodriguez-Garcia

**Affiliations:** Centro de Física Aplicada y Tecnología Avanzada, 73403 Universidad Nacional Autónoma de México, Campus Juriquilla, Querétaro, Querétaro 76230, Mexico

## Abstract

Biogenic hydroxyapatite (BHA) exhibits physicochemical variability associated with donor-dependent factors. BHA extracted from young and adult Duroc-Jersey porcine bones was systematically compared, with a focus on predominant substitutional ions and crystallite size. BHA powders were obtained by controlled thermal treatment. Thermal analysis by TGA and DSC revealed age-dependent differences in thermal stability and coalescence behavior related to variations in organic content and carbonate species. ICP-OES showed nonstoichiometric compositions with Ca/P ratios and trace-element concentrations, particularly silicon-containing species, which differed significantly between the samples investigated. FT-IR spectroscopy confirmed the formation of AB-type carbonated hydroxyapatite and silicate substitutions. HR-TEM revealed crystallite sizes within the transition range between nanometric and micrometric regimes (60–100 nm). XRD showed lattice distortions consistent with multi-ionic substitution. These results suggest that differences in ionic composition between the young- and adult-derived samples are associated with variations in structural organization and thermal behavior.

## Graphical abstract

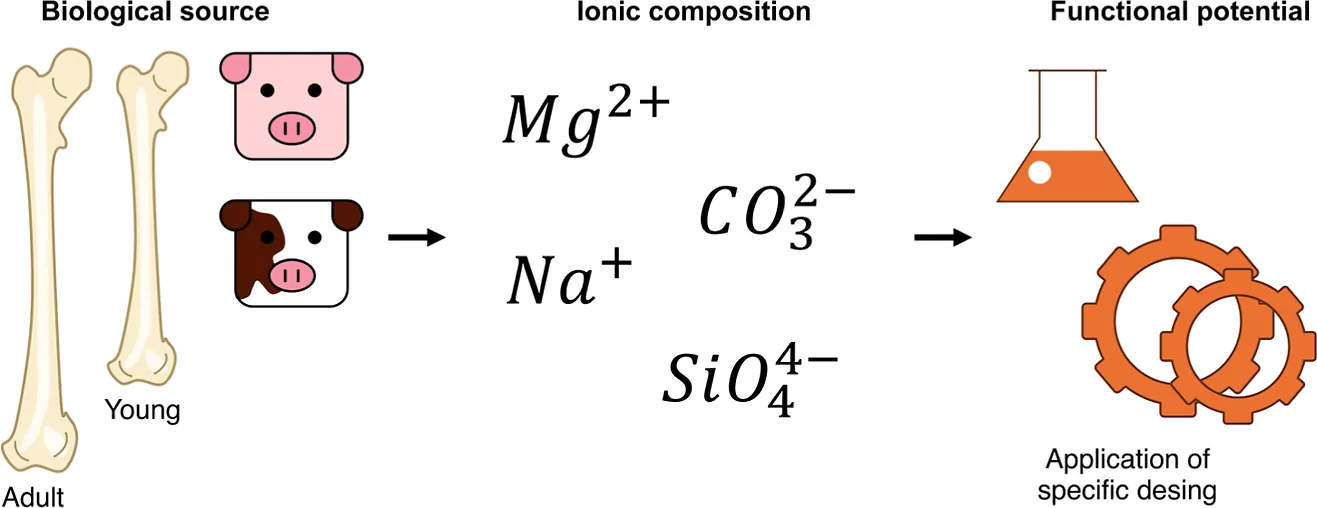

## 1 Introduction

Hydroxyapatite (HA) is among the most studied calcium phosphate ceramics because of its significance in biomedical applications, catalysis, and environmental remediation.^1–3^ Its physicochemical properties, such as vibrational modes, crystallite size (τ), and chemical composition, including substitutional ions in the crystal lattice, play important roles in various scientific and engineering applications.^1^ These variables can be especially significant when HA is derived from natural sources (biogenic hydroxyapatite, BHA), such as mammalian bone, where factors like sex and life stage may influence its properties.

Several ceramic biomaterials have been investigated for applications in bone regeneration and tissue engineering. Alumina-based bioceramics exhibit excellent chemical stability, hardness, wear resistance, and long-term durability, making them attractive for load-bearing orthopedic applications.^4,5^ Zirconia-based biomaterials have also received considerable attention because of their high fracture toughness, mechanical strength, chemical stability, and favorable biocompatibility. In particular, yttria-stabilized zirconia has been widely used in dental restorations, while zirconia-based ceramics have progressively evolved from high-performance structural materials into multifunctional biomaterials for orthopedic and dental applications.^6–8^ However, despite their excellent mechanical performance, alumina and zirconia are generally considered bioinert and do not closely replicate the mineral composition of natural bone. In contrast, hydroxyapatite has a chemical composition similar to the inorganic phase of bone tissue, promoting osteo conductivity, bioactivity, and improved integration with biological systems.^1^ Therefore, understanding the physicochemical factors governing hydroxyapatite remains essential for the development of advanced biomaterials for bone-related applications.^1^

HA can be produced through synthetic chemical routes that achieve a Ca/P ratio close to the theoretical value (1.66) or can be intentionally doped with selected elements. When HA is obtained by these methods, temperature control is essential for its stability, morphologic, crystalline phase, and other properties.^9,10^ Studies confirm that ionic incorporation, whether intentional or incidental, significantly modifies its physicochemical behavior.^9^

When HA is extracted from biological sources such as organic waste from pigs,^11^ fish,^12^ horses,^13^ and other sources,^15^ it is called biogenic hydroxyapatite (BHA). The impact of this material is due to the different substitutional ions in its crystal structure, which can enhance its performance compared with synthetic HA in various applications. As with synthetic HA, for BHA, it is important to control the thermal conditions during processing to preserve the nanometric scale, since factors such as large plateaus, nonquasi-static processes, cooling atmosphere, and furnace characteristics such as heat capacity affect the resultant material.^11,12^ Bone maturity significantly influences crystallinity and elemental composition.^13^ Therefore, the stage of life plays an important role in how BHA behaves during applications, while sex affects the concentration of different ions, as gestational processes influence bone mineral density.^13–15^ Based on this, one could argue that organic waste from males is better for extracting BHA.

Intrinsic multi-ionic substitution provides a better framework for understanding this material. Among these ions are Mg^2+^, Na^+^, CO_3_^2–^, and SiO_4_^4–^, along with trace elements such as Zn^2+^, Ag^+^, and Li^+^, all of which affect lattice symmetry and defect concentration.^16,17^ Although studies such as those by Mugnaioli et al.^17^ have considered these factors, they often do not account for the actual occupancy of each substitutional ion based on experimental results, nor do they consider other characteristics of the source, such as the specimen sex. Similarly, Londoño Restrepo et al.^14^ showed through in situ high temperature X-ray diffraction that calcination induces progressive crystallite growth and structural ordering in cattle-derived HA. Complementary work by Chakraborty et al.^18^ demonstrated that both synthetic and biogenic apatites undergo measurable microstructural evolution upon heating, manifested in peak narrowing and changes in lattice parameters. More recently, Querido et al.^19^ correlated ultrastructural organization with mineral phase evolution in bone-like matrices, emphasizing the sensitivity of apatite crystallinity to biological and processing variables. These studies clearly establish the importance of thermal history; however, they lack a comparative analysis based on substituent ions and sample variability. Although different factors, such as donor age and sex, may indirectly modify the ionic composition of BHA, particularly in systems subjected to metabolic demands, there is still a lack of systematic experimental correlations between biological age, trace element content, and physicochemical behavior.

From a theoretical standpoint, first-principles calculations have been used to investigate the response of HA to ionic substitution. Ren et al.^20^ used density functional theory to analyze the elastic and structural effects of carbonate substitution in apatite, providing insight into lattice distortion mechanisms. In a complementary study, Zilm et al.^21^ combined experimental and theoretical approaches to examine transition metal substitution in HA, demonstrating that isolated ionic substitutions can significantly alter structural stability. However, these theoretical frameworks typically consider single ion substitutions under idealized conditions and lack direct comparison with experimentally refined occupancies obtained from X-ray diffraction. Consequently, their applicability to real biogenic systems, where multiple substitutional ions coexist, remains limited.

Crystallite size is another key parameter influencing the functional performance of HA. In biomedical applications, nanometric or sub-100 nm crystallites are often preferred because of enhanced bioactivity and interfacial response. Kumar and Agrawal demonstrated that controlling HA crystal size in composite systems can significantly reduce stress shielding effects in bioimplant applications.^22^ In contrast, Amor et al.^23^ showed that nanocrystalline HA exhibits superior performance in environmental applications such as heavy metal adsorption, where surface area and defect density dominate functional behavior. These studies highlight that crystallite size and ionic composition must be considered together to optimize application specific performance.

Despite extensive work on BHA, comparative analyses that explicitly consider donor age, experimentally measured substitutional ion concentrations, and crystallographic refinement remain limited, particularly for porcine derived systems. Porcine bone is an attractive model due to its chemical similarity to human bone and its availability as organic waste; however, the impact of differences in ionic composition between young- and adult-derived porcine BHA has not been comprehensively addressed.

Although BHA has been extensively investigated, studies that simultaneously correlate donor age, experimentally measured ionic composition, thermal behavior, crystallite-size evolution, and crystallographic refinement remain scarce. In particular, the influence of age-dependent species on the structural organization of porcine-derived hydroxyapatite has not been systematically examined. The novelty of the present work lies in establishing direct relationships between biological age, multi-ionic substitution, and crystallographic response in hydroxyapatites extracted from controlled Duroc-Jersey porcine sources.

This study investigate the physicochemical differences between biogenic hydroxyapatite obtained from adult and young male porcine bones of the Duroc-Jersey breed, with the use of male specimens minimizing variations associated with gestational effects on bone mineral density. The research focuses on substitutional ions experimentally detected at concentrations exceeding 100 ppm and correlates their presence with crystallographic parameters obtained from X-ray diffraction and Rietveld refinement. Particular attention is given to materials with crystallite sizes in the transition range between nanometric and micrometric regimes (60 nm ≤ τ ≤ 100 nm), providing insight into how differences in ionic composition observed between the investigated young- and adult-derived samples influence structural organization, thermal behavior, and potential application-driven performance.

## 2 Materials and Methods

### 2.1 Sample Obtention

#### 2.1.1 Origin of the Bones

Bones were obtained from young and adult male pigs of the Duroc-Jersey breed, slaughtered at 119 days (73.450 kg) and 196 days (203.102 kg), respectively. The pigs were raised on a controlled diet under the climatic conditions of San Juan del Río, Querétaro, Mexico.^24^ Feeding followed Table 1, using Albapesa feed. During the first 3 weeks of life, they were fed prestarter Fine Wean 0. After that, they received the following prestarter feeds: Fine Wean 1 from 6 to 9 kg, Fine Wean 2 from 9 to 12 kg, Fine Wean 3 from 12 to 15 kg, and Fine Wean 4 from 15 to 30 kg. They were then fed a grower diet from 30 to 69 kg with Concentrado de Crecimiento. Finally, finisher diets were provided as follows: from 69 kg to slaughter for young pigs and up to 85 kg for adult pigs with ALB Concentrado de Engorda, while adults weighing more than 85 kg until slaughter received ALB de Engorda Magro. Apple and alfalfa intake began at the same time as the prestarter diets and continued until slaughter. The pigs were castrated during the second week of life.

**Table 1:** Percentage of the Guaranteed Analysis by Albapesa^25^

food	minimum protein (%)	minimum of raw fat (%)	minimum crude fiber (%)	maximum humidity (%)	maximum ash (%)
prestarted					
fine wean 0	21	7	3	7	12
fine wean 1	21	7	4	7	12
fine wean 2	20	5	6	7	12
fine wean 3	20	5	6	7	12
fine wean 4	20	4	5	8	12
growing food					
Concentrado de Crecimiento	40	1.5	12	18	12
finisher food					
ALB Concentrado de Engorda	38	1.5	8	20	20.5
ALB Concentrado de Engorda Magro	40	1.5	10	19	12

#### 2.1.2 Bone Powder Preparation

The raw pig bone powder was prepared following the methodology of Bernal-Alvarez et al.^11^ First, the cortical bone was cut into 5 cm thick slices using a meat saw. The slices were boiled in distilled water for 1 h and then dried in an Ecocell oven at 90 °C for 1 h to remove fat and remaining soft tissue (organic material). Next, the slices were cleaned by a hydrothermal process in a Terlab autoclave at 120 °C and 103 kPa. This process was repeated five times to eliminate any residual organic material. Finally, the slices were crushed and ground using an RS 300 vibrating Disc Mill by Retsch to obtain particles smaller than 75 μm (200 mesh). The raw powder was labeled Y-RAW and Ad-RAW for young and adult pigs, respectively. It is important to note that the raw bone powder was obtained from the cortical bone of the diaphysis of both femurs from the same specimen, with one specimen used for the adult samples and another for the juvenile samples.

#### 2.1.3 BHA Powder Preparation

The BHA powder with the desired crystallite size was obtained by calcining the raw bone powder at 800 °C with a heating rate of 5 °C/min, a 10 min plateau, and cooling in air to room temperature, as reported by Bernal-Alvarez et al.^11^ The samples were named Y-BHA for young porcine and Ad-BHA for adult porcine.

### 2.2 Thermal Analysis

Raw samples were analyzed using thermogravimetric analysis (TGA) and differential scanning calorimetry (DSC) to determine material degradation, BHA coalescence, and sintering temperatures. TGA was performed with a TA Instruments model T500 in air. DSC was conducted with a Mettler Toledo TGA/DSC 2 STARe system in air. For both analyses, 9.0 ± 0.5 mg of material was heated from 27 to 1000 °C at 5 °C/min.

### 2.3 Elemental Composition Analysis

Inductively coupled plasma optical emission spectroscopy (ICP-OES) was used to quantify minerals and determine the Ca/P ratio. An iCAP PRO Duo argon plasma device from Thermo Fisher Scientific was used for this analysis. Samples were digested with 7 mL nitric acid (Baker 69.3%) and 0.1 g of each sample, then filtered using Whatman No. 42 filters for filtration. Samples were analyzed in duplicate.

### 2.4 Vibrational Analysis

A FT-IR Spectrum Two PerkinElmer spectrophotometer with a Zn–Se diamond in the spectral range from 600 to 4000 cm^–1^, in air, 4 cm^–1^ resolution, 64 scans and Attenuated Total Reflectance (ATR) accessory was used to study the vibrational modes of BHA.

### 2.5 Experimental Crystallite Size Analysis by HR-TEM

High-resolution transmission electron microscopy (HR-TEM) was used to examine the atomic arrangements of sintered BHA. A 1:1 mixture of deionized water and isopropyl alcohol was used to dissolve the samples, which were then dispersed with ultrasound. A sample droplet was placed in the holder using a capillary system. Images of the samples were taken with a Jeol HR-TEM, the ARM200F. The open-source ImageJ software was used to calculate the crystallite sizes of the Ad-BHA sample from the bright and dark field images.

### 2.6 Structural Analysis

The structural analysis of the Y-BHA and Ad-BHA samples was carried out by X-ray diffraction. The data was obtained from a conventional Rigaku Ultima IV powder X-ray diffractometer with a Bragg–Brentano configuration, equipped with a copper tube (CuK_α_ radiation wavelength λ = 1.5406 Å) and a graphite monochromator (002), in a range from 5° to 80° in 2θ, with 0.01° increments, a scanning speed of 2°/min, at ambient temperature and humidity, and a sample holder rotation speed of 15 rpm.

At the same time, ICP-OES data were used to estimate the maximum theoretical substitution fractions of the detected ionic species (eq 1). These values were employed as chemically informed constraints during the construction of the structural model for Rietveld refinement. Given the low concentrations of minor substituents, particularly silicon-containing species, these fractions were not independently determined from the diffraction data.

1 $$F_{i}=\frac{ppm}{pm\times n_{related}}≔1.00$$

where *F*_*i*_ represents the relative substitution fraction of the *i*-th species, ppm is its concentration determined by ICP-OES, *pm* is the molecular weight of the corresponding ionic species, and *n*_related_ corresponds to the relative molar amount used for normalization. The obtained values represent the maximum fraction of substituting ions chemically available in the material and were subsequently used as constraints during the Rietveld refinement procedure.

Relative substitution fractions obtained from the ICP-OES results were used to define the maximum allowable occupancies of the corresponding crystallographic sites during refinement. Subsequently, Rietveld refinement was then performed using Profex 5 V 5.2.^26^ The occupancies reported in the structural model, therefore represent crystallographic parameters constrained by the experimentally determined chemical composition.

The instrumental contribution to the broadening of the diffraction peaks was corrected using a standard LaB_6_ sample (Standard Reference Material 660c) from the National Institute of Standards and Technology (NIST).

The diffraction profiles were modeled using a Thompson-Cox-Hastings (TCH) pseudo-Voigt function, which provides an analytical approximation to the actual Voigt profile and allows for the consistent separation of the Gaussian and Lorentzian contributions associated with the material microstructure and the instrumental function.^27–29^ For these reasons, the average crystallite size was calculated using a modification of Scherrer equation that contains the microstrain with an anisotropic model.^22,30^

## 3 Results and Discussion

### 3.1 Thermal Analysis

Figure 1a shows the mass loss of bone raw powder (Y-RAW and Ad-RAW) samples as a function of temperature and the second derivative. The second derivative is used to identify the inflection points of the graph, which mark changes in concavity and thus indicate the initial and final points of mass loss in a given region. The first mass loss (zone 1), where 1% of the mass is lost, is due to dehydration from adsorbed water. The second mass loss (zone 2) is related to the loss of organic material and crystallization water.^31^ The third mass change (zone 3) results from the partial loss of lipids and proteins.^31^ The complete removal of resistant proteins in BHA corresponds to the fourth concavity change (zone 4).^31^ The fifth mass drop (zones 5 and 6) corresponds to the loss in weight of BHA, specifically associated with carbonate ions.^31^ Zone 7 is due to the dehydroxylation of BHA.^16^

**Figure 1:**
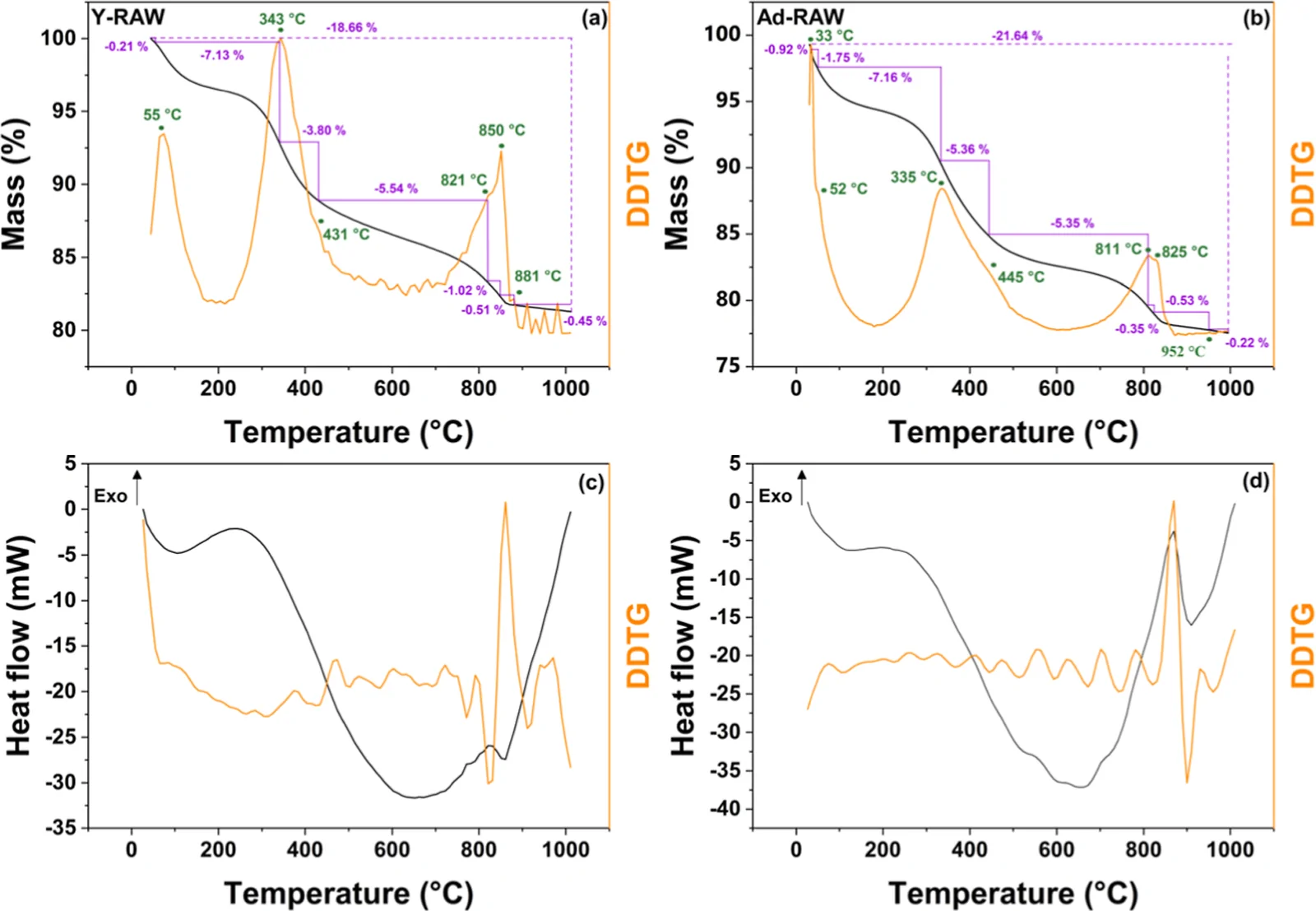
(a,b) Thermogravimetric analysis of bone raw powder (Y-RAW and Ad-RAW). (c,d) Differential scanning calorimetry of sintered samples (Y-BHA and Ad-BHA).

Several known thermal events related to BHA are observed in the DSC (Figure 1b). The first thermal events in zone 1 correspond to the release of H_2_O absorbed by BHA.^32^ Similarly, events in zone 2 are due to the release of H_2_O bound to BHA and the decomposition of organic matter, specifically collagen as well as the initiation of the degradation of BHAs own carbonate groups.^16,33^ In zone 3, each thermal event corresponds to the partial loss of CO_3_^2–^ groups.^16,34^ In zone 4, proteins are degraded, breaking their primary structure,^35,36^ and the four main endothermic events in hydroxyapatite are generated, as reported in the literature, namely the four coalescence events of BHA.^34,35^ The first is known as low-temperature coalescence, which is interplanar, surface, and superficial; that is, the crystallographic planes begin to coalesce, and according to Cañon-Davila et al.,^33^ this occurs at around 400 °C. The next two, at approximately 500 and 600 °C, respectively, increase the crystal size, but keep it within the nanometer range.^37^ The last event is reported at around 720 °C where HA increases in size and exceeds the nanometer regime (<100 nm).^14^ However, Bernal-Alvarez et al.^11^ recently performed an in situ study and found a nanometer crystal size (>100 nm) when biogenic hydroxyapatite is sintered up to 800 °C with a short plateau, then cooled in air to avoid stress in the crystal lattice. This prevents the final endothermic event from occurring, so the change in crystal size does not take place in zone 4 but instead between zones 5 and 6. Thus, the reported coalescence is not due to the temperature reached but to the duration of the plateau during sintering, as a greater amount of energy is supplied to the system. In previous reports, this plateau lasts for hours.^14,33,37^

In zone 6, the thermal events correspond to magnesium diffusion from the BHA, caused by the characteristic substituent ions of the bile source,^35^ which generates a leaching effect as described by Ramirez-Gutierrez et al.^36^ At the upper temperature limit of zone 6 and in zone 7, dehydroxylation of HA begins due to the high temperatures,^32^ resulting in the transformation of hydroxyapatite into oxyapatite.^37^ Therefore, based on the above, the sintering temperature for the sample was set at 800 °C as reported by Bernal-Alvarez et al.^11^

### 3.2 Elemental Composition Analysis

BHA has a nonstoichiometric composition due to biomineralization and the presence of various trace elements, resulting from factors such as diet, age, and health. For these reasons, the Ca/P ratios of Y-BHA and Ad-BHA are 1.64 and 1.62, respectively (Table 1). This indicates that the samples contain different elements, as shown in Table 2. However, some of these are trace elements, present only in minimal amounts (>100 ppm), such as Al (25 ppm), B (90 ppm), Ba (9 ppm), Cr (10 ppm), Cu (2 ppm), Fe (153 ppm), K (424 ppm), Li (48 ppm), Mn (3 ppm), Ni (6 ppm), S (425 ppm), Sr (88 ppm), V (13 ppm), and Zn (267 ppm).

**Table 2:** Elemental Composition in ppm and Ca/P Ratio of Sintered Sample Using ICP-OES

element	Y-BHA	Ad-BHA
Ca	425372 ± 10	423106 ± 11
Mg	12638 ± 16	12736 ± 18
Na	6161 ± 15	6396 ± 12
P	165243 ± 20	170058 ± 15
Si	395 ± 2	2146 ± 5
Ca/P	1.64	1.62

Each mineral contributes to bone composition, especially calcium and phosphorus. Calcium maintains serum levels, strengthens bones, and is important for blood and immune system function,^14^ while phosphorus, present as PO_4_^3–^ groups, is the second most abundant mineral constituent of bone.^38^ P plays a role in bone formation and regulates proliferation, differentiation, and mineralization in osteoblasts and preosteoblasts as well as apoptosis.^14^ In addition, substitution ions that are not trace elements, also play an important role in the bone system. Boron is important for bone regeneration, bone growth, and the prevention of osteoporosis, although is still needed for the latter.^39^ Boron is present in bone as BO_3_^3–^ or BO_2_^–^ ions.^40^ Iron (Fe^2+^/Fe^3+^) is important for tissue regeneration and its deficiency leads to poor mineralization of osteoblast cells.^41^ Magnesium, in the form of Mg^2+^, is responsible for bone growth and osteogenesis in the early stages and is also key to the regulation of osteoblasts and osteoclasts, proliferation, cell differentiation, and bone metabolism.^16^ For biomineralization to occur, sodium is essential because Na promotes mineralization, which irrigates the site of bone formation.^9^ Sulfur (as SO_4_^2–^), stimulates bone growth.^42^ Silicon, present as SiO_4_^4–^ groups in the BHA lattice, improves osteoblast activity, regenerative properties, and bone formation and growth.^43^ Strontium (as Sr^2+^) is important for the bone remodeling process and is found in low concentrations.^44^ Zinc, in the form of Zn^2+^ ions, is essential for the normal function of more than 80 enzymes, making it an important substitution ion in bone.^45^

### 3.3 IR Analysis

Table 3 shows the vibrational modes of the raw and sintered samples shown in Figure 2, identified using the second derivative methodology reported by Bernal-Alvarez et al.^46^ The OH^–^ group identified in band A corresponds to the structural hydroxyl group of HA, whereas band B is attributed to water absorbed from the environment due to the hydrophilic nature of the material.^37^ The latter is less pronounced than band A because the H_2_O molecules, although vibrating at the same frequency, are randomly distributed in the material, causing phononic overlaps. The presence of organic compounds, attributable to fats and proteins, corresponds to the source of BHA extraction. Collagen was identified by bands C and D.^47,48^ Band E refers to amide I, while band F is the superposition of the N-Hδ_s_ and C–H νs of amide II and amide III. The carbonates present in the samples (bands G, H and N) are substitutional in the lattice, locally bound to Na and a channel (type A) or to a phosphate (type B), creating defects across the ion.^49^ A-type carbonate substitutes the hydroxyl ion on the *c*-axis.^49^ The literature also notes that the B-type carbonate is located at an angle of 53° with respect to the specular plane on one of the oblique faces of the phosphate.^49^

**Table 3:** Vibrational Mode Identification of all Samples^a^ ^c^

	wavenumber (cm^–1^)	vibrational mode assignment	samples	refs
A	3559	O–H ν_s_ (HA)	^b^,^b^	^50,53^
B	3250	O–H ν_s_ of H_2_O absorption	^b^	^50,53^
C	2919	C–H of CH_2_ν_as_	^b^	^50,53^
D	2852	C–H of CH_2_ ν_*s*_	^b^	^54^
E	1640	C=O ν_s_ of amide I	^b^	^48^
F	1547	N-Hδs y C–H ν_*s*_ (amide II and amide III)	^b^	^16,47^
G	1448	CO_3_^2–^ type A ν_3as_	^b^,^b^	^49^
H	1414	CO_3_^2–^ type B ν_3as_	^b^,^b^	^49^
I	1096	PO_4_^3–^ ν_3as_	^b^,^b^	^10,51,53^
J	1017	PO_4_^3–^ ν_3as_	^b^,^b^	^10,51,53^
K	960	PO_4_^3–^ ν_1s_	^b^,^b^	^10,51,53^
L	878	Si–O ν_s_ of SiO_4_^4–^	^b^	^52^
M	870	CO_3_^2–^ type B ν_2s_	^b^,^b^	^10,51,53^
N	631	OH^–^ ν_s_	^b^	^10,51,53^

a Y-RAW and Ad-RAW.

b Y-BHA and Ad-BHA.

c ν: stretching; δ: bending.

**Figure 2:**
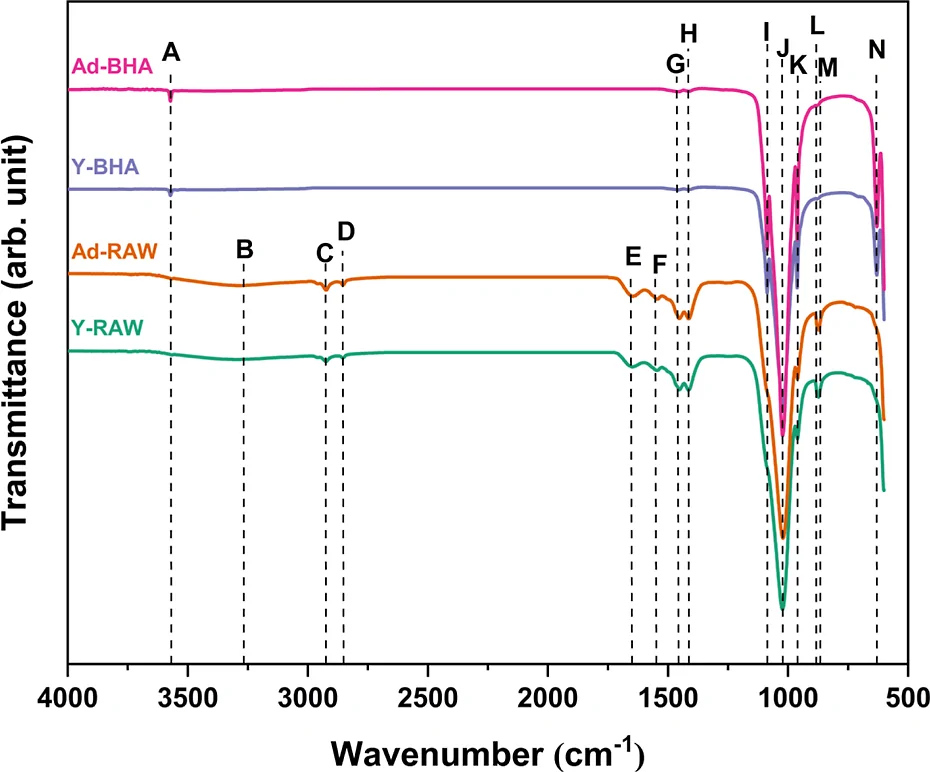
IR spectra before and after the thermal process of all samples.

PO_4_^3–^ is the typical form of the phosphate group in apatite.^50^ This functional group is difficult to analyze in the solid aggregate state because it can exist in different forms depending on its bonding. In BHA, the tribasic orthophosphate ion has C_1_ symmetry which is simplified by *T*_d_ symmetry.^51^ For this reason, the degenerate triplet stretching mode (ν_3as_) is unequally split.^51^ The K-band corresponds to the fully symmetrical vibration after energy interaction. The L band corresponds to the silicate anion,^40,52^ due to ionic substitutions within the BHA, given the nature of the material since it was extracted from porcine bone.

The transmittance in the N-band is described as a combination of two components resulting from Fermi resonance, which causes partial band splitting; the band divides into two parts: one at a higher frequency and the other oscillating around a center at 620 cm^–1^.^50^

### 3.4 Experimental Crystallite Size Analysis

Figure 3 shows HR-TEM images of Y-BHA and Ad-BHA samples at a scale of 200 nm. The hydroxyapatite shows agglomerates and semicircular plates, consistent with previous studies.^9,16^ In Ad-BHA, the crystallites were entirely within the nanometric range, with average sizes of 70.48 ± 17.20 nm for Y-BHA and 61.91 ± 10.81 nm for Ad-BHA, respectively (Figure 3a,b,f and g). This finding is consistent with previous reports by Aminian et al.^54^ and Arcos et al.,^55^ which suggest that silicate incorporation may contribute to limiting crystallite growth in hydroxyapatite. However, the present results only establish an association between the higher silicon content detected in Ad-BHA and the observed reduction in crystallite-size. After assessing the normality of both groups using the Shapiro–Wilk test, no significant deviation from normality was observed (Y-BHA: *W* = 0.936, *p* = 0.333; Ad-BHA: *W* = 0.949, *p* = 0.276). Since Levene’s test indicated unequal variances (*F* = 12.654, *p* = 0.001), Welch’s *t* test was applied. The analysis showed no statistically significant difference between the Y-BHA and Ad-BHA groups (*t* = −0.693, df = 31.91, *p* = 0.493). The effect size was small (Cohen’s *d* = −0.20), indicating that the difference between the group means was negligible. It should be noted that this statistical analysis was performed on crystallite-size measurements obtained from multiple crystallites in the HR-TEM images and therefore evaluates differences between crystallite populations rather than biological replicates.

**Figure 3:**
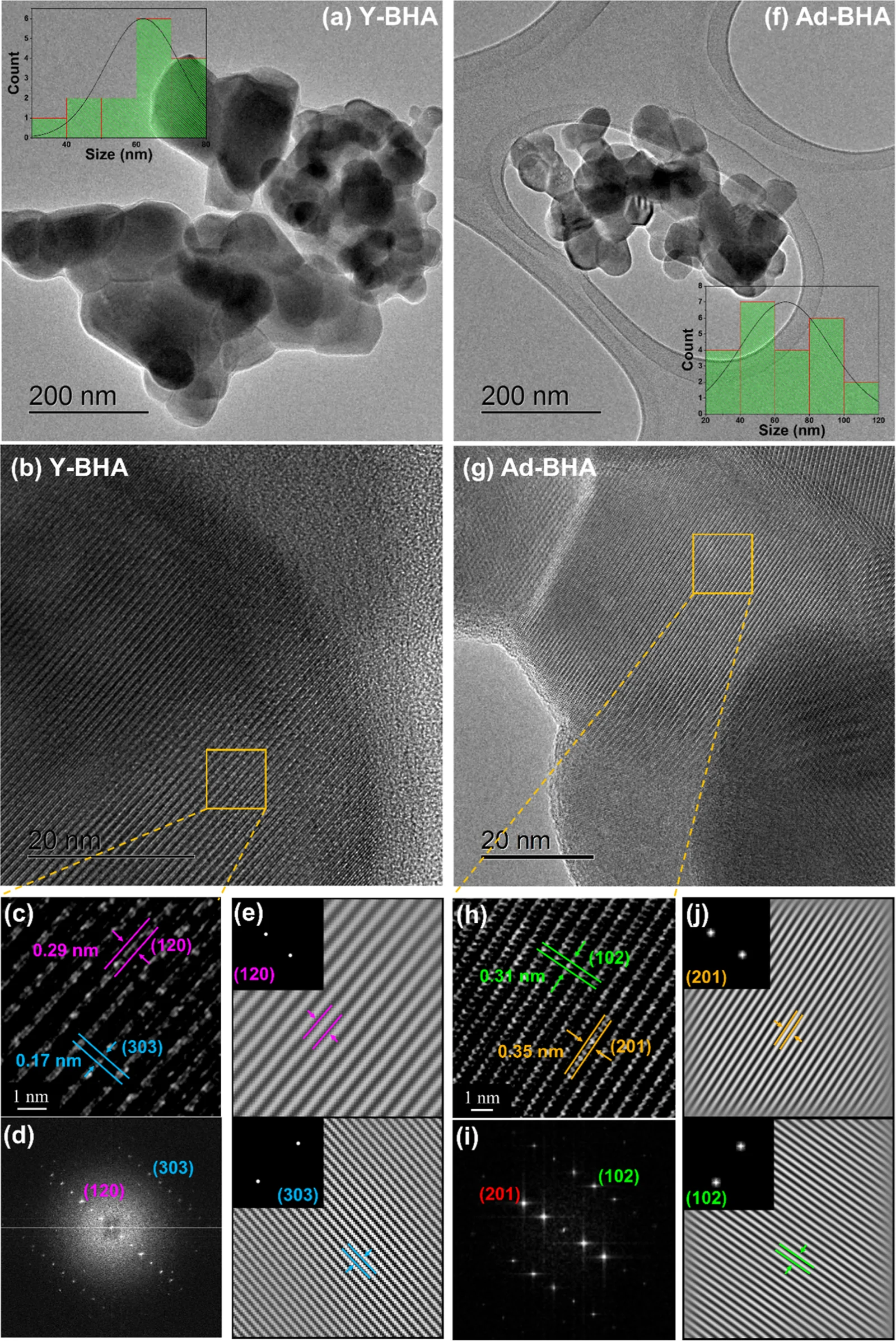
(a,b) HR-TEM image at a scale of 200 nm with the corresponding histogram, showing the particle size distribution of Y-BHA. An image at a scale of 20 nm is also presented, including: a magnified view of the selected region (area marked in yellow), the Fourier transform of that region, and the inverse Fourier transform of the specific area. (c–e) Details of these images are shown for sample Y-BHA. (f,g) HR-TEM image at a scale of 200 nm with the corresponding histogram, showing the particle size distribution of Ad-BHA. An image at a scale of 20 nm is also presented, including: a magnified view of the selected region (area marked in yellow), the Fourier transform of that region, and the inverse Fourier transform of the specific area. (h–j) Details of these images are shown for sample Ad-BHA.

Although the cleaning process reduces the amount of organic matter, as described in the thermal analysis, this organic matter is known to surrounding the BHA nanocrystals. Therefore, the nanocrystals begin to grow earlier because the organic matter is removed sooner, resulting in a longer period during which the crystals are fully exposed to the incident energy in the system.

Additionally, the sample exhibits polycrystallinity, as several crystalline planes are observed in the Fast Fourier Transform (FFT) (see Figure 3c–e and h–j). Analysis with Gatan Digital Micrograph V 4.0 software revealed the presence of the (120), (303), (102), and (201) crystalline planes with interplanar distances of 0.29, 0.17, 0.31, and 0.35 nm, respectively.

### 3.5 Structural Analysis

For the structural analysis of BHA extracted from young and adult porcine sources, it is important to consider the predominant ions in the compound and the IR spectra of the sintered sample. In this case CO_3_^2–^, Na^+^, Mg^2+^, and SiO_4_^4+^ are considered only as substituted ions in the crystal lattice (with the silicon cation present only in the Ad-BHA sample). The F_i_ from the ICP-OES results are shown in Table 4. These values were used as chemical constraints during refinement and should not be interpreted as occupancies independently determined from the diffraction data, in which the only element with a concentration greater than 1% was Mg^2+^. This indicates that, given the resolution of the equipment, the BHA samples are unlikely to contain significant amounts of Na^+^ or Si^4+^, with the latter present only in the Ad-BHA sample (Table 4). Therefore, Rietveld refinement was performed, as shown in Figure 4.

**Table 4:** F_*i*_ of Y-BHA and Ad-BHA Samples

element	Y-BHA	Ad-BHA
Mg	0.0112	0.0113
Na	0.0006	0.0007
Si	0.0003	0.0018

**Figure 4:**
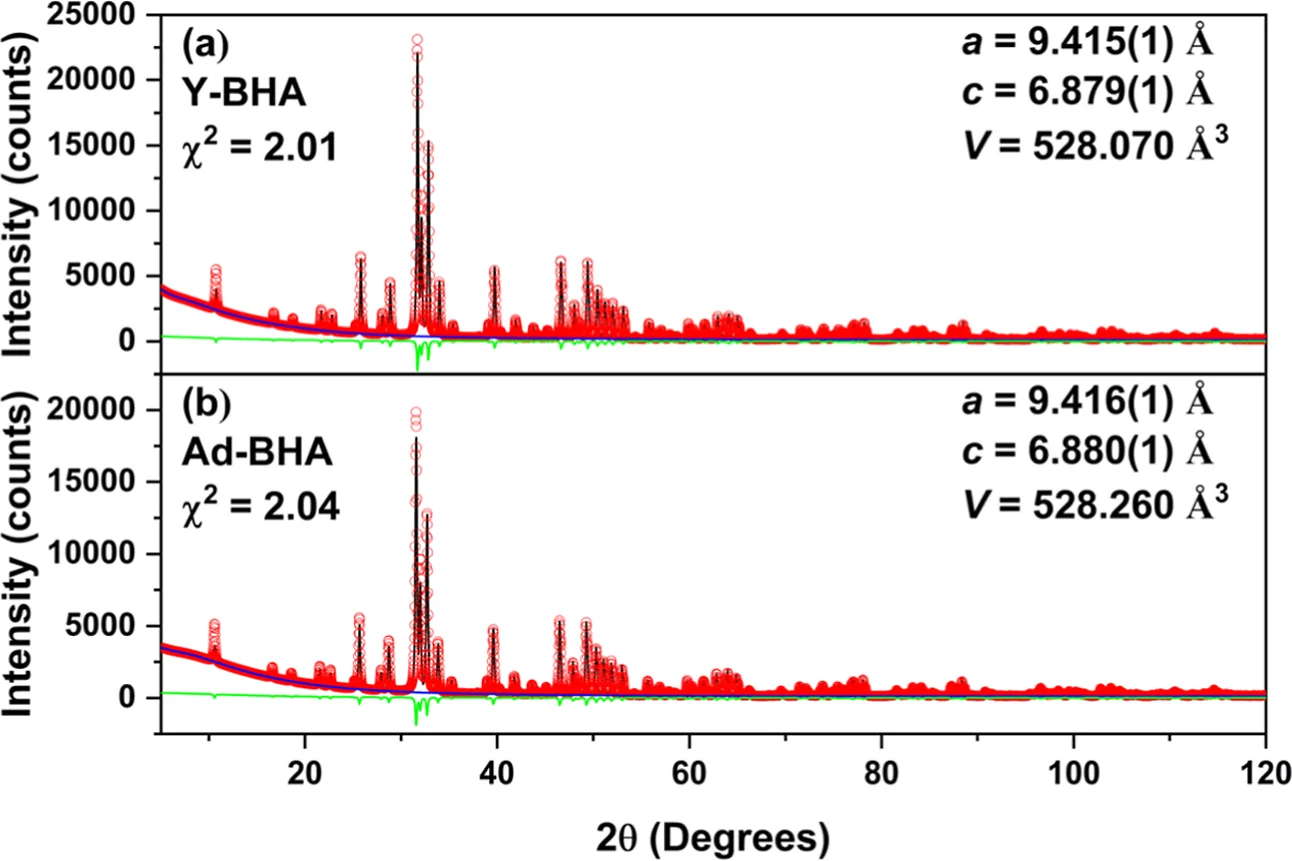
Rietveld refinement of (a) Y-BHA and (b) Ad-BHA.

It is important to note that, although ab initio and DFT studies indicate that sodium and magnesium substitution is favorable at the Ca­(I) position,^16,23^ in this model the preferred sites are Ca­(I) and Ca­(II), respectively. This could be due to a diet rich in silicon, as the ion enters in silicate form (SiO_4_^4–^). This results in a chemical formula described as $Ca_{\left(10-x-\gamma \right)}Mg_{x}Na_{y}{\left(PO_{4}\right)}_{\left(6-\xi -\zeta \right)}{\left(SiO_{4}\right)}_{\xi }{\left(CO_{3}\right)}_{\zeta }{\left(OH\right)}_{2}$ , with the same crystal angles as a hexagonal lattice (α = β = 90° and γ = 120°), but with variations in lattice parameters and unit cell volume compared to synthetic HA (see Table 5). The difference between the results of Sudarsanan & Young^56^ and the present sample is due to the presence of silicate and carbonate ions.

**Table 5:** Lattice Parameters and Volume Cell (*V*) of Ad-BHA and the Reported by Sudarsanan & Young^56^

	Y-BHA	Ad-BHA	reference^a^
*a*(Å)	9.415(1)	9.416(1)	9.424(4)
*c*(Å)	6.879(1)	6.880(6)	6.876(4)
*V*_*c*_(Å^3^)	528.070	528.260(17)	529.090

a Reported by Sudarsanan & Young.^52^

The average crystallite sizes in the [110], [111], and [002] directions were 72.20 ± 0.50, 72.69 ± 0.37, and 77.77 ± 0.96 nm for Y-BHA and 66.73 ± 0.47, 67.39 ± 0.35, and 68.87 ± 0.32 nm for Ad-BHA, values that are similar to the averages measured by HT-TEM. Because Mg^2+^ and Na^+^ concentrations remain nearly constant in both samples, their contribution is expected to affect the overall structural organization of biogenic hydroxyapatite rather than the differences observed between Y-BHA and Ad-BHA. The most significant compositional difference is associated with the silicon-containing species detected in Ad-BHA, whose relative substitution fraction is approximately six times higher than that in Y-BHA. Therefore, the reduction in crystallite size and the subtle variations in lattice parameters observed for Ad-BHA may be associated with the higher concentration of silicon-containing species, consistent with previous reports on silicon-substituted hydroxyapatites.^55,56^

Although Mg^2+^ and Na^+^ did not differ significantly between the two samples, they remain important because they participate in charge-compensation mechanisms and contribute to the overall structural stabilization of the apatite lattice. Mg^2+^, with an ionic radius of 0.086 nm compared to 0.099 nm for Ca^2+^, promotes a local contraction of the coordination environment, increasing electrostatic interactions and favoring lattice stabilization. In contrast, Na^+^, whose ionic radius (0.102 nm) is slightly larger than that of Ca^2+^, tends to increase local interatomic distances, promoting lattice relaxation and influencing the *c* lattice parameter. Consequently, while Mg^2+^ and Na^+^ contribute to structural characteristics common to both materials, the differences observed between Y-BHA and Ad-BHA are more reasonably attributed to the higher silicate content in the adult-derived hydroxyapatite.

It should be noted that SiO_4_^4–^ and PO_4_^3–^ differ in ionic radius, with the former measuring 0.026 nm and| the latter 0.017 nm. This difference causes changes in the lattice parameters, particularly along the *a*-axis and not along c, as it directly affects the formed polyhedron.^55–57^

Carbonate ions, with an ionic radius of 0.016 nm, replace phosphate and hydroxyl ions, resulting in AB-type carbonated BHA, the most energetically stable form, as demonstrated by Ren et al.^20^ This substitution occurs in similar proportions within the crystal lattice and is promoted by Na^+^ in the Ca­(I) site, which reduces the amount of Mg^2+^, unlike BHA, which does not have AB-type carbonate substitution.^17^ Without these ions, the compound would be less stable, as noted by Ressler et al.^16^ and Ren et al.,^20^ and the size along the *c*-axis would increase because Na^+^ directly affects this direction.

## 4 Conclusions

A physicochemical comparison of BHA extracted from one young and one adult Duroc-Jersey pig raised under controlled feeding and climatic conditions revealed physicochemical differences between the young- and adult-derived samples. Thermal analysis by TGA and DSC shows that the coalescence phenomenon in BHA depends not only on the sintering temperature but also on the thermal residence time. This indicates that the weight loss and thermal events observed in the young and adult BHA raw powder samples are associated with differences in the concentrations of the composite compounds, resulting in crystallite evolution from the nanometric to the micrometric scale. Elemental analysis (ICP-OES) revealed that BHA is nonstoichiometric, as the Ca/P ratios differ from the theoretical value of 1.66 for HA. This variation is attributed to age and sex, which are closely related to bone maturity and directly influence charge-compensation mechanisms within the apatite lattice, particularly those involving *g* ^2+^, Na^+^, and silicon-containing species. These differences may be associated with metabolic and dietary factors related to bone maturity and directly affect charge-compensation mechanisms within the apatite lattice.

IR spectroscopy confirms the presence of AB-type carbonated hydroxyapatite in both samples, as well as vibrational features associated with silicate substitution. These results support the coexistence of multiple substitutional mechanisms in porcine-derived BHA, which contribute to lattice distortion and structural stabilization. HR-TEM revealed crystallite sizes within the transition range of 60–100 nm for both samples. The lower average crystallite size observed in adult-derived BHA coincided with a higher concentration of silicon-containing species; however, the crystallite-size differences were not statistically significant. Therefore, the present results indicate an association rather than a demonstrated causal effect of silicate substitution on crystallite-growth behavior.

Structural analysis confirms an increase in the lattice parameter along the *c*-axis and a decrease along the *a*-axis associated with the presence of various substituent ions within the unit cell, which tend to stabilize the structure. This is consistent with reports in the literature. The incorporation and variation of these ions promote the formation of AB-type carbonated hydroxyapatite, in which charge balance is achieved through the presence of species such as Na^+^, Mg^2+^, and SiO_4_^4–^.

It should be noted that the present study was conducted using one young and one adult specimen. Therefore, the observed differences should be interpreted as characteristics of the investigated samples rather than population-level age effects. Additional studies including independent biological replicates are necessary to establish statistically robust age-dependent trends in porcine-derived biogenic hydroxyapatite.

Overall, the results obtained for the investigated samples indicate that BHA with these physicochemical properties has increased potential for specific applications, especially where the presence of substitutional ions, and control of crystallite size are key factors in obtaining customized materials.

## Data Availability

Data will be made available on request.
